# Old Ironsides

**DOI:** 10.14797/mdcvj.1214

**Published:** 2023-03-07

**Authors:** James B. Young

**Affiliations:** 1 Emeritus Executive Director of Academic Affairs, Cleveland Clinic and Professor Emeritus of Medicine, Cleveland Clinic Lerner College of Medicine of Case Western Reserve University, Cleveland, Ohio, US; 2 Section Editor, Poet’s Pen, Methodist DeBakey Cardiovascular Journal

## Abstract

Dr. Oliver Wendell Holmes Sr. was a 19th century elite physician, curmudgeon, essayist, and poet. His works were numerous, insightful, entertaining, and characteristic of the pre-Civil War, Civil War, and Gilded age eras. Many of his shorter poems are taught in high school as examples of late 19th century works, as they are relatively easy to memorize and understand. Holmes and the Fireside Poets (Emerson, Longfellow, and Lowell, among others) created works that, in their time, were read aloud by fathers and mothers to their family because the poems often centered around values, morals, and historic events. “Old Ironsides” is characteristic of the type of work for which the Fireside Poets became famous. It is an entertaining poem to read, study, and hear, particularly when linking it to the oldest ship in the world, still afloat and sailing around Boston Bay.

## Old Ironsides

Ay, tear her tattered ensign down!

    Long has it waved on high.
And many an eye has danced to see
    That banner in the sky;
Beneath it rung the battle shout,
    And burst the cannon’s roar; —
The meteor of the ocean air
    Shall sweep the clouds no more!

Her deck, once red with heroes’ blood
    Where knelt the vanquished foe,
When winds were hurrying o’er the flood
    And waves were white below,
No more shall feel the victor’s tread,
    Or know the conquered knee; —
The harpies of the shore shall pluck
    The eagle of the sea!

O, better that her shattered hulk
    Should sink beneath the wave;
Her thunders shook the mighty deep,
    And there should be her grave;
Nail to the mast her holy flag,
    Set every thread-bare sail,
And give her to the god of storms, —
    The lightning and the gale!

Oliver Wendell Holmes Sr, MD

## Uss Constitution as “Old Ironsides”

“Old Ironsides”^1,2^ is an iconic poem written by the physician, curmudgeon, and poet Oliver Wendell Holmes Sr (1804-1894). Holmes Sr should not be confused with his son, Oliver Wendell Holmes Jr (1841-1934), associate justice of the Supreme Court, nicknamed “the Great Dissenter.”

The poem has been a staple for youthful classes of literature and poetry because of its catchy tone, cadence, and important reference to American history. Its style is old school and short enough to be easily memorized (three eight-line stanzas), with a catchy rhyming pattern as each stanza has its own distinct rhyming scheme.^1,2,3^ It was written in 1830, on the eve of the warship USS Constitution being decommissioned from the United States Navy, and very early in the career of Holmes as a poet and curmudgeon. The poem was published in the *Boston Daily Adviser* in response to an article in the *New York Journal of Commerce*, detailing the fact that the USS Constitution was to be decommissioned and sundered for scrap. This was after an extraordinary history of protecting democracy and the United States of America. Particularly notable was its defense during the War of 1812.

**Figure 1:**
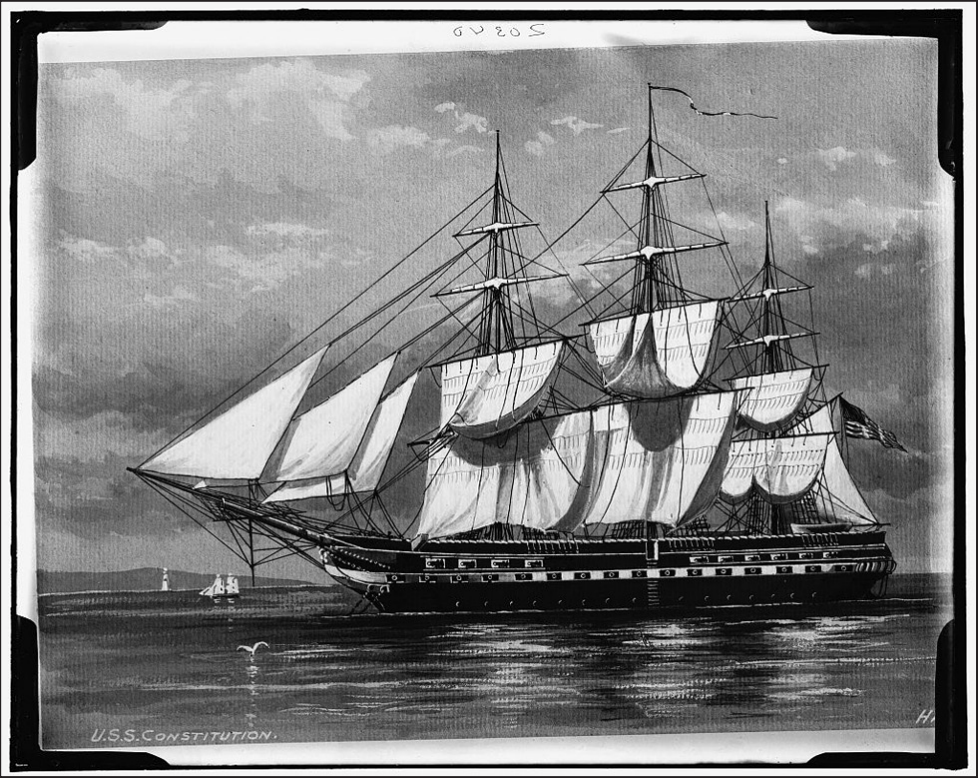
*USS Constitution port broadside*. [Between 1900 and 1920] Photograph. Detroit Publishing Co., Publisher. Retrieved from the Library of Congress, <www.loc.gov/item/2016817278/>

This is the first poem in Holmes’ poetry compendium after his solicitous introduction to his readers.^2^ At the beginning of the poem, Holmes seems to take the position of endorsing decommissioning of this glorious warship. The poem suggests that the distinctive ensign, that was flown high, should be torn down and permanently fixed to the ship’s mast. The author had other ideas about how to honor the glory of the historic USS Constitution by putting it on display, albeit that “…her shattered hulk/should sink beneath the wave” but only after nailing “to the mast her holy flag” and setting “every thread-bare sail” while giving her to the god of storms.

The history of the USS Constitution goes back to the beginning of our republic. She is a three-masted heavy frigate with an impenetrable oak hull (thus the name “Ironsides”), launched in 1797 as part of the Naval Act of 1794. She was the third of six commissioned warships. George Washington himself approved the name “Constitution.” She protected merchant shipping during conflicts with France and the First Barbary War. Her actions in the War of 1812 were particularly significant and important, prompting public adoration that saved her from the scrap heap on several occasions.

The oldest ship in the world still floating, and intermittently sailing, she currently is on “active duty,” moored in Boston Harbor near the Boston Freedom Trail with a Navy crew of around 75 sailors. My attachment to the ship is the name of the Ohio Township, Bainbridge, in which I live. When my wife and I recently toured the USS Constitution, I discovered that Captain William Bainbridge was the frigate’s commander during the War of 1812. Several other municipalities, many streets, and a few warships carry the name “Bainbridge” as well. I have yet to come across any neighbors who know the history of our township’s name or know much about the War of 1812 and how our Ohio “Western Reserve” got the name and why.

**Figure 2:**
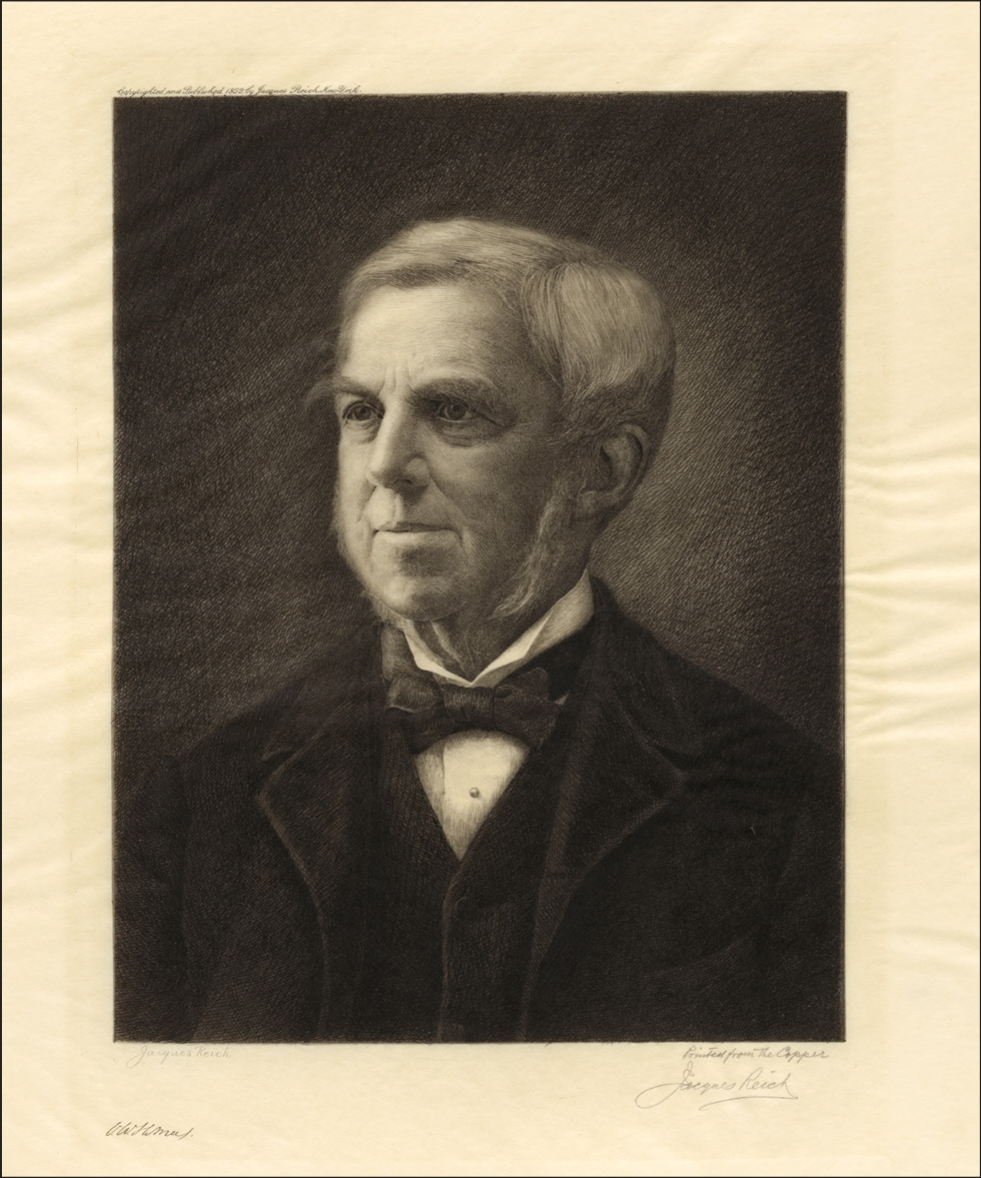
Oliver Wendell Holmes Sr, by Jacques Reich, 1899. National Portrait Gallery, Smithsonian Institution; gift of Oswald D. Reich.

Oliver Wendell Holmes Sr studied medicine in Paris at first, then returned to Boston and Harvard School of Medicine, where he received his medicine degree in 1836 after he penned “Old Ironsides.”^4^ He taught at Dartmouth School of Medicine before returning to Boston and Harvard, where he had a long and distinguished career. He championed the unpopular concept of puerperal fever being an infection transmitted by doctor’s carrying the scourge from patient to patient via filthy hands, clothing, and instruments.

Holmes also was part of a literary group of prominence that included Henry Wadsworth Longfellow, William Cullen Bryant, John Greenleaf Whittier, and James Russell Lowell, collectively known as the Fireside Poets. His works were often published in *The Atlantic Monthly*, and he continued his literary career throughout his medical career, earning him the label of a literary physician writer (William Carlos Williams was of the same ilk almost a century later).

With respect to the profession of medicine, Holmes was a reformer. In addition to his attention to puerperal fever, he focused on the poor hygienic conditions of the Boston Dispensary. He railed against many contemporary practices with the quote, “If all contemporary medicine was tossed into the sea it would be all the better for mankind—and all the worse for the fishes.”^5^ He also demeaned quackeries and homeopathy, labeling the later “the pretended science…mingled with a mass of perverse ingenuity, of tinsel erudition, of imbecile credulity, and of artful misrepresentation, too often mingled in practice.”^6^

Holmes traveled extensively throughout New England, wrote poetry, essays, and novels, saw patients, and at one time was critical of the abolitionists. In the end, he was a passionate supporter of the Union side of the Civil War. Later in life, Holmes slowed as he aged but also traveled to Europe, received numerous honorary degrees, kept up his association with his literary friends, and died peacefully in 1894. He seems better known for his literary adventures than his medical work. Though his poetry and other writings might be labeled stilted and old fashioned, careful reading uncovers his wit and wisdom.
